# The Composition of the Cuticular and Internal Free Fatty Acids and Alcohols from *Lucilia sericata* Males and Females

**DOI:** 10.1007/s11745-012-3662-5

**Published:** 2012-03-14

**Authors:** Marek Gołębiowski, Mieczysława I. Boguś, Monika Paszkiewicz, Wioletta Wieloch, Emilia Włóka, Piotr Stepnowski

**Affiliations:** 1 Institute for Environmental and Human Health Protection, Faculty of Chemistry, University of Gdańsk, ul. Sobieskiego 18/19, 80-952 Gdańsk, Poland; 2 Institute of Parasitology, Polish Academy of Sciences, Twarda 51/55, 00-818 Warsaw, Poland

**Keywords:** Cuticular and internal lipids, HPLC–LLSD, GC–MS, *Lucilia sericata*, *Conidiobolus coronatus*, Fungal infection

## Abstract

GC, GC–MS, and HPLC–LLSD analyses were used to identify and quantify cuticular and internal lipids in males and females of the blow-fly (*Lucilia sericata*). Sixteen free fatty acids, seven alcohols and cholesterol were identified and quantitatively determined in the cuticular lipids of *L. sericata*. Cuticular fatty acids ranged from C_6_ to C_20_ and included unsaturated entities such as 16:1n-9, 18:1n-9, 20:4n-3 and 20:5n-3. Cuticular alcohols (only saturated and even-numbered) ranged from C_12_ to C_20_ in males and C_10_ to C_22_ in females. Only one sterol was found in the cuticular lipids of both males and females. 23 free fatty acids, five alcohols and cholesterol were identified in the internal lipids. Internal fatty acids were present in large amounts—7.4 mg/g (female) and 10.1 mg/g (male). Only traces of internal alcohols (from C_14_ to C_26_ in males, from C_14_ to C_22_ in females) were found in *L. sericata*. Large amounts of internal cholesterol were identified in *L. sericata* males and females (0.49 and 0.97 mg/g of the insect body, respectively).

## Introduction

*Lucilia* species (Calliphoridae), important pollinators of flowering plants, are distributed worldwide and are the best known species in human infestation in America, Africa, and Asia. These ectoparasites are found in the meat and corpses of animals, and cause myiasis in humans and domestic herbivorous animals [1–3]. In 1826, myiasis caused by *L. sericata* in humans was reported by Magen. It was then that the parasites were isolated from the mouth, eyes, and paranasal sinuses of a hospital patient for the first time.

Unlike the larvae of some other myiasis-causing flies, *L. sericata* larvae rarely invade living healthy tissues surrounding a necrotic wound. Due to this fascinating phenomenon, called facultative myiasis, *L. sericata* larvae have been used since antiquity as a safe and effective wound treatment. The secretions of maggots are known to stimulate in vitro increase in total human fibroblasts [4] and have antibacterial properties [5]. Although no in vivo reports regarding wound healing mechanisms of maggot therapy are available, recent data show that fly cuticular fatty acids may play a significant role in this process. Fatty acid extracts of dried *L. sericata* larvae called in traditional Chinese medicine “WuGuChong” and used to treat superficial purulent diseases such as furuncle or carbuncle, can promote murine cutaneous wound healing probably resulting from the powerful angiogenic activity of the extracts [6].

The cuticle of all insects is covered with a very thin epicuticular layer of wax. The cuticle consists of several layers, from the outside to the inside: the epicuticle, the procuticle and the epidermis. The insect cuticle is the first barrier against biological or chemical contact insecticides; it is mostly resistant to enzyme degradation and exhibits characteristic water barrier properties [7].

Naturally occurring entomopathogens are important regulatory factors of insect populations. The potential use of fungal pathogens to control insects is well documented, and the use of fungi as control agents against insect pests has been reviewed [8–12]. Susceptibility or resistance of various insect species to fungal invasion may result from several factors, including differences in the structure and composition of the exoskeleton, the presence of antifungal compounds in the cuticle, as well as the efficiency of cellular and humoral defense reactions of the invaded insect [13]. It is believed that the epicuticular lipid profile of the insect host may be one of pivotal factors determining insect susceptibilities or resistance to fungal attack [14]. The mode of action of entomopathogenic fungi involves the attachment of fungal spores to the cuticle, followed by spore germination and, depending on the fungal species, formation of appressorium or penetrative hyphae. Penetration is then initiated, involving both mechanical and enzymatic mechanisms [15]. Once inside the host, the fungus propagates, consuming nutrients and releases metabolites (some of which might be toxic), which results in mycosis and, ultimately, host death [9, 10].

The ability of the fungus to fully degrade the epicuticular hydrocarbon components of its insect host, utilizing them as an exogenous carbon source, was demonstrated by Napolitano and Juarez [16]. The presence of a wax layer potentially affects spore germination by fungilytic or fungistatic toxicity, or by acting as a barrier to the chitin matrix of the insect exoskeleton, effectively preventing the spore from coming into contact with nutrients or other cues that trigger germination. Thus, the waxy layer produced on the cuticle may act as a first line of defense against fungal pathogens. Little information on both, stimulatory and inhibitory effects of cuticular fatty acids on growth and virulence of insecticidal fungi is available. Medium- and short-chain fatty acids and alcohols have been demonstrated to be toxic to filamentous fungi, including some that have been isolated specifically from insect cuticle [17, 18], but the toxicity of long-chain fatty acids is unknown. Kerwin [19] demonstrated the toxic effects of 6:0, 7:0, 9:0, 10:0, 18:2 and 18:3 fatty acids on *Entomophthora culicis* conidia. However, 18:1 was found to have a positive effect on spore germination and to mitigate the harmful effects of 18:3 acid. The concentration of fatty acids also had an important impact on fungal growth and conidia development: 0.1% 16:1 was toxic to secondary conidia, but positively affected fungal growth. Cuticular lipids were found to have toxic or inhibitory effects on the conidia of *B. bassiana* and *P. fumosoroseus* when the spores were germinated on nutrient agar in the presence of lipids [19]. Eighteen fatty acids identified in the larval cuticle of three insect species representing differing susceptibilities to *Conidiobolus coronatus* infection [20], were thoroughly tested for effects on the in vitro growth and pathogenicity of this parasitic fungus [21].

This paper describes the cuticular lipid composition of *L. sericata* adults. The surface lipids of flies were separated into classes of compounds using HPLC–LLSD. Qualitative and quantitative analyses were done by GC and GC–MS. The determination of the composition of fly lipids and their impact on the development and pathogenicity of *C. coronatus* may have great practical importance and will allow using this fungus or its metabolites to control insect populations.

## Materials and Methods

### Insects

*Lucilia sericata* raised from eggs laid on beef by adult flies, were reared at 25 °C with 50% relative humidity and a 12:12 h photoperiod. Maternal generation was maintained in the same conditions. The insects were fed on beef and it took them approximately 7 days from hatching to puparium formation and another 7 days to the appearance of adult. The insects were exposed for 18 h to fully grown and sporulating fungal colonies. Ten flies were kept in each Petri dish (males and females separately). The adults exposed for 18 h to sterile uninoculated Sabouraud agar supplemented with *G. mellonella* larval extract served as a control. After exposure, the insects were transferred to clean Petri dishes with sugar and water and kept under their growing conditions for 10 days. The condition of the exposed animals was monitored daily. Exposure of tested insects to a *C. coronatus* colony for 18 h was found to be most efficient method resembling the natural infection process [22]. In order to avoid pseudo replication, all assays of fungi vs. insects were performed with the use of flies from different stocks incubated in three different chambers.

A culture of the wax moth, *Galleria mellonella* was maintained and reared in temperature and humidity controlled chambers (30 °C, 70% r.h.) in constant darkness on an artificial diet [23]. Fully grown larvae were collected before pupation, surface sterilized, homogenized and used as a supplement in fungal cultures.

### Fungus

*Conidiobolus coronatus*, isolate number 3491, originally isolated from *Dendrolaelaps spp*., was obtained from the collection of Prof. Bałazy (Polish Academy of Sciences, Research Center for Agricultural and Forest Environment, Poznań), routinely maintained in 90-mm Petri dishes at 20 °C with cyclic changes of light (L:D 12:12) on Sabouraud agar medium (SAM) with the addition of homogenized *Galleria mellonella* larvae to a final concentration of 10% wet weight. Addition of homogenized *G. mellonella* larvae enhances SAM cultures of *C. coronatus.* The levels of mycelial growth, conidia production, and virulence were good in hundreds of successive transfers [24] suggesting a stimulatory effect of insect proteins, carbohydrates and lipids on *C. coronatus* growth and insecticidal properties.

### Extraction of Insects

**Fig. 1:**
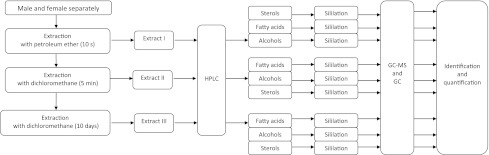
Scheme of the analysis

Figure 1 shows the scheme for preparing the sample and analysis. Male and female specimens of *L. sericata* were extracted first in petroleum ether for 10 s and then a second time in dichloromethane for 5 min [20]. These two extracts (petroleum extract I and dichloromethane extract II) contained cuticular lipids. The third extraction was a long one with dichloromethane for 10 days (III extract). This third extract contained internal lipids. Then, 0.5 ml of the whole extract was placed in a glass flask and then evaporated under nitrogen to determine the dry mass of the remaining extracted lipids. Table 1 lists the number of adult insects, as well as the masses of the extracts.

**Table 1:** Quantitative summary of the experiment: numbers and masses of insect; masses of lipids

	Number of insects	Insects (g)	Extracts	Lipids in extracts (mg)	Lipids	
					(mg/insect)	(mg/g of the insect body)
Male	75	1.7	I	5.1	0.07	3.0
			II	1.3	0.02	0.8
			III	42.3	0.56	24.9
Female	80	2.3	I	3.2	0.04	1.4
			II	1.6	0.02	0.7
			III	45.4	0.57	19.7

I petroleum extract (10 s)

II dichloromethane extract (5 min)

III dichloromethane extract (10 days)

### High Performance Liquid Chromatography

All lipid extracts of the males and females (I, II and III) were separated into several classes of compounds by HPLC in the normal phase using a Shimadzu LP-6A binary pump in gradient mode. A laser light scattering detector was used as the detection system [25]. To obtain large amounts of lipids for GC–MS analysis the separation was repeated five times. All fractions were evaporated, silylated and analyzed by GC and GC–MS.

### Gas Chromatography

The investigations were carried out on a Clarus 500 (Perkin Elmer) gas chromatograph equipped with a Rtx 5 fused silica column (30 m × 0.25 mm i.d., film thickness 0.1 μm) was used. The oven temperature was held at 80 °C for 10 min and then increased at a rate of 4 °C/min to a final temperature of 320 °C [25].

### Gas Chromatography–Mass Spectrometry

Gas chromatography–mass spectrometry measurements were carried out by coupling an SSQ 710 (Finnigan Mat) spectrometer to a Hewlett-Packard 5890 gas chromatograph. The samples were introduced through the gas chromatograph equipped with a 30 m × 0.25 mm i.d., Optima-5 silica capillary column and with a 0.25 μm thick film. The oven temperature 80 °C (held for 10 min) was increased to 320 °C at 4 °C/min. The injector temperature was 320 °C and the carrier gas was helium. The ion source was maintained at 220 °C.

The lipids were derivatized by methods described previously [20]. All compounds were identified by comparing the retention time of the analyzed compounds with standards and on the basis of silyl derivative ions. The mass spectrum of trimethylsilyl ethers of fatty acids showed the following ions: M^+·^ (molecular ion), [M−15]^+^, and fragment ions at *m*/*z* 117, 129, 132, and 145. Cholesterol was identified on the basis of the characteristic ions of silyl derivatives (*m*/*z* 458 (M^+·^), 129, 329, 368, 145, 121 and 353). Characteristic ions of alcohols (trimethylsilyl derivatives) were [M−15]^+^ and *m*/*z* 103 [26].

### Statistical Analysis

In order to quantitatively determine each of the compounds analyzed, GC analysis was performed with internal standards (19-methylarachidic acid, 1-octacosanol and sitosterol). Data are presented as the means ± standard deviations of three separate analyses performed on different samples. The data obtained were statistically processed by using the *t* pairwise test for determining significant differences in males and females lipids concentration.

## Results

### Susceptibility of *L. sericata* to Fungal Infection

Exposure of *L. sericata* adults to the sporulating *C. coronatus* resulted in the prompt death of all tested males and females (Table 2). Both, males and females died around the termination of the 18-h exposure to the fungal culture. The lack of resistance of *L. sericata* males and females on *C. coronatus* infection are confirmed by results obtained with the *t* pairwise test. Based on the *t* pairwise test we did not notice any significant differences in males and females susceptibility to fungal infection.

**Table 2:** The susceptibility of *Lucilia sericata* males and females to fungal infection

Developmental stage/treatment	Number of insects	Mortality (%)^a^
Adult females		
Control	30	0
Exposed to *C. coronatus*	50	100
Adult males		
Control	47	0
Exposed to *C. coronatus*	50	100

^a^Adult insects were exposed to sporulating *C. coronatus* colonies as described in the "Materials and Methods" section. The insect susceptibility to fungal infection is expressed as percentage of mortality in tested populations

### Extraction of *L. sericata* Lipids

This is the first time that the chemical composition of the cuticular and external lipids in the *L. sericata* has been analysed and identified. Three extractions of male and female *L. sericata* were performed. The petroleum ether (I) and dichloromethane (II) extracts of males yielded 3.0 and 0.8 mg/g of the insect body, respectively. These two extracts consisted of cuticular lipids, so the quantity of lipids amounted to 3.8 mg/g of the insect body. The quantities of male internal lipids yielded 24.9 mg/g of the insect body (0.56 mg/insect). The quantities of cuticular lipid extracts obtained from female *L. sericata* were smaller than those from the males and amounted 2.1 mg/g of the insect body (1.4 and 0.7 mg/g of the insect body in extracts I and II, respectively). Also, the quantities of female internal lipids were less than in the males and yielded 19.7 mg/g of the insect body, but the quantity of internal lipids per insect was almost the same (0.57 mg/female vs. 0.56 mg/male).

Extracts of lipids from male and female *L. sericata* were separated by HPLC–LLSD into fractions containing general groups of chemical entities. The lipids extracted from the males and females contained four fractions: hydrocarbons, triacylglycerols, free fatty acids (FFA) and sterols. The triacylglycerols were present in the internal lipids of male and female (III extracts). They serve as a source of energy stored in fat body. The hydrocarbons were present in cuticular lipids (I and II extracts) in small amounts. They can play an important role as pheromones. In this study were determined compounds with potential antimicrobial activity, including the free fatty acid, alcohol and sterol fractions. These fractions were further analyzed by GC and GC–MS. For qualitative purposes, the instrument was operated in total ion current (TIC) mode and single ion monitoring (SIM) mode for monitoring [M−15]^+^ ions (Table 4). Single ion monitoring mode was used to achieve high selectivity and sensitivity. Gas chromatography was used for the quantitative analyses of lipids. Comparisons were made with the lipids extracted from male and female *L. sericata*.

### Fatty Acid Composition of Male *L. sericata*

Table 3 summarizes the results obtained during the identification and quantification of FFA in the cuticular lipids of male *L. sericata*. The two cuticular extracts (petroleum ether-I and dichloromethane-II) contained 15 compounds from C_6_ to C_20_. The dominant cuticular fatty acids (all with an even number of carbon atoms) were: 16:1n-9 (10.3%), 16:0 (29.4%), 18:1n-9 (40.6%) and 18:0 (6.7%). The total free fatty acid content in cuticles in males was 14.3 μg/g (6.1 μg/g in the petroleum ether extract and 8.2 μg/g in the dichloromethane extract).

**Table 3:** Chemical composition of the cuticular fatty acids found in males and females of *Lucilia sericata*

FFA	Content (μg/1 g)		Relative content % (w/w)	
	Cuticular FFA (male)	Cuticular FFA (female)	Cuticular FFA (male)	Cuticular FFA (female)
6:0	0.47 ± 0.04	0.14 ± 0.02	3.3	1.6
7:0	–	0.15 ± 0.01	–	1.7
8:0	0.12 ± 0.02	0.12 ± 0.01	0.8	1.4
9:0	0.45 ± 0.03	0.32 ± 0.04	3.1	3.6
10:0	0.14 ± 0.02	0.14 ± 0.01	1.0	1.6
11:0	Traces	Traces	Traces	Traces
12:0	0.26 ± 0.03	0.28 ± 0.03	1.8	3.2
14:1n-9	–	–	–	–
14:0	0.31 ± 0.03	0.31 ± 0.03	2.2	3.5
15:0	0.14 ± 0.01	0.15 ± 0.02	1.0	1.7
16:1n-9	1.48 ± 0.07	1.34 ± 0.06	10.3	15.2
16:0	4.2 ± 0.2	2.8 ± 0.1	29.4	31.8
17:1n-10	–	–	–	–
17:0	Traces	–	Traces	–
18:2n-6	–	–	–	–
18:1n-9	5.8 ± 0.4	2.2 ± 0.1	40.6	25.0
18:0	0.96 ± 0.05	0.70 ± 0.05	6.7	8.0
19:0	–	–	–	–
20:5n-3	Traces	0.09 ± 0.01	Traces	1.0
20:4n-3	Traces	0.14 ± 0.02	Traces	1.6
20:1n-6	–	–	–	–
20:0	–	Traces	–	Traces
22:0	–	–	–	–
24:0	–	–	–	–
26:0	–	–	–	–
Sum	14.3	8.8	–	–

Cuticular FFA: sum of FFA content in petroleum extract (I), and dichloromethane extract (II)

Data are presented as the means ± standard deviations of three separate analyses performed on different samples

The internal lipids of male *L. sericata* contained 21 FFA (Table 4). The total free fatty acid content in the internal lipids of the male was 10.1 mg/g of the insect body. The fatty acids occurring in the highest concentrations were 16:1n-9 (14.7%), 16:0 (19.5%) and 18:1n-9 (57.4%). The fatty acids 17:1n-10, 18:2n-6, 19:0, 20:1n-6, 20:0, 22:0, 24:0 and 26:0 occurred only in the internal lipids. On the other hand, 10:0 and 11:0 acids occurred only in the cuticular lipids.

**Table 4:** Chemical composition of the internal fatty acids (extract III) found in males and females of *Lucilia sericata*

FFA	Content (μg/1 g)		Relative content % (w/w)		[M−15]^+^ Monitored ions (SIM mode)
	Internal FFA (male)	Internal FFA (female)	Internal FFA (male)	Internal FFA (female)	
6:0	1.8 ± 0.1	1.33 ± 0.05	<0.1	<0.1	173
7:0	–	0.40 ± 0.02	–	<0.1	187
8:0	0.86 ± 0.04	0.96 ± 0.05	<0.1	<0.1	201
9:0	0.59 ± 0.03	0.40 ± 0.02	<0.1	<0.1	215
10:0	–	Traces	–	Traces	229
11:0	–	–	–	–	243
12:0	10.4 ± 0.8	11.0 ± 0.5	0.1	0.1	257
14:1n-9	–	Traces	–	Traces	283
14:0	16.4 ± 0.9	13.1 ± 0.5	0.2	0.2	285
15:0	1.9 ± 0.1	2.0 ± 0.1	<0.1	<0.1	299
16:1n-9	14.8 × 10^2^ ± 0.5 × 10^2^	12.9 × 10^2^ ± 0.3 × 10^2^	14.7	17.5	311
16:0	19.7 × 10^2^ ± 0.6 × 10^2^	15.5 × 10^2^ ± 0.5 × 10^2^	19.5	21.0	313
17:1n-10	6.1 × 10^1^ ± 0.3 × 10^1^	3.9 × 10^1^ ± 0.2 × 10^1^	0.6	0.5	325
17:0	8.2 ± 0.4	6.6 ± 0.5	0.1	0.1	327
18:2n-6	2.6 × 10^1^ ± 0.1 × 10^1^	14.7 ± 0.6	0.3	0.2	337
18:1n-9	5.8 × 10^3^ ± 0.2 × 10^3^	39.3 × 10^2^ ± 1 × 10^2^	57.4	53.2	339
18:0	22.8 × 10^1^ ± 0.9 × 10^1^	16.5 × 10^1^ ± 0.7 × 10^1^	2.3	2.2	341
19:0	Traces	Traces	Traces	Traces	355
20:5n-3	4.2 × 10^2^ ± 0.1 × 10^2^	29.3 × 10^1^ ± 0.7 × 10^1^	4.2	4.0	359
20:4n-3	13.8 × 10^1^ ± 0.4 × 10^1^	8.9 × 10^1^ ± 0.3 × 10^1^	1.4	1.2	361
20:1n-6	Traces	1.23 ± 0.08	Traces	<0.1	367
20:0	6.5 ± 0.3	4.6 ± 0.2	0.1	0.1	369
22:0	1.59 ± 0.06	0.97 ± 0.06	<0.1	<0.1	397
24:0	4.7 ± 0.2	3.0 ± 0.2	<0.1	<0.1	425
26:0	0.81 ± 0.03	0.61 ± 0.03	<0.1	<0.1	453
Sum	10.1 × 10^3^	73.9 × 10^2^	–	–	–

Data are presented as the means ± standard deviations of three separate analyses performed on different samples

### Fatty Acid Composition of Female *L. sericata*

The cuticular lipids of female *L. sericata* contained 16 FFA from C_6_ to C_20_ (Table 3). Ten FFA from C_8_ to C_18_ were identified in the petroleum ether extract (I) and all 16 fatty acids from C_6_ to C_20_ in the dichloromethane extract (II). The total free fatty acid content in the cuticle in females was 8.8 μg/g (2.0 μg/g in the petroleum ether extract and 6.8 μg/g in the dichloromethane extract). The percentage contents of fatty acids were very diverse (from traces to 31.2%). The FFA occurring in the highest concentrations were 16:1n-9 (15.2%), 16:0 (31.8%), 18:1n-9 (25.0%) and 18:0 (8.0%).

More fatty acids were present in the internal lipids; they ranged from C_6_ to C_26_ (Table 4) (like the internal lipids in the males). The total free fatty acid content in the internal lipids of the female was 7.4 mg/g of the insect body. Twenty four saturated, monounsaturated and polyunsaturated fatty acids were present in the internal lipids. Saturated (16 compounds) and monounsaturated fatty acids (five compounds) were dominant. The FFA occurring in the highest concentrations were 16:1n-9 (17.5%) 16:0 (21.0%) and 18:1n-9 (53.2%). A similar profile of the major compounds was identified in the cuticular lipids, except that the major compound was 18:1n-9 in the internal lipids and 16:0 in the cuticular lipids. The following acids present in the internal lipids were absent from the cuticular lipids: 14:1n-9, 17:1n-10, 17:0, 18:2n-6, 19:0, 20:1n-6, 22:0, 24:0 and 26:0. On the other hand, only 11:0 was absent from the internal lipids of female *L. sericata*, whereas the cuticular lipids contains traces of it.

### Alcohol Composition of Male *L. sericata*

Table 5 lists the percentage contents of alcohols in the cuticle as well as the alcohol contents calculated per g of insect body. Only five alcohols were found in the cuticular lipids of the males and ranged from C_12:0_ to C_20:0_. The compound present in the highest concentrations was C_18:0_ (0.37 μg/g of the insect body; relative content 55.2% of total alcohols). The total cuticular alcohol content in male *L. sericata* was only 0.67 μg/g. Only traces of five alcohols were found in the internal lipids. These alcohols have more carbon atoms than the cuticular ones, from C_14:0_ to C_26:0_. The internal lipids contain traces of three alcohols (C_22:0_, C_24:0_ and C_26:0_), which were absent from the cuticular lipids. All the identified alcohols were saturated, with even-numbered carbon chains.

**Table 5:** Chemical composition of the alcohols found in male of *Lucilia sericata*

Content (μg/1 g)					Relative content % (w/w)	
Alcohols	Cuticular alcohols (male)	Internal alcohols (male)	Cuticular alcohols (female)	Internal alcohols (female)	Cuticular alcohols (male)	Cuticular alcohols (female)
C_10_	–	–	Traces	–	–	Traces
C_12_	0.05 ± 0.01	–	0.11 ± 0.01	–	7.5	13.8
C_14_	0.13 ± 0.01	Traces	0.13 ± 0.01	Traces	19.4	16.3
C_16_	Traces	–	0.03 ± 0.01	–	Traces	3.8
C_18_	0.37 ± 0.03	–	0.39 ± 0.03	–	55.2	48.8
C_20_	0.12 ± 0.01	Traces	0.14 ± 0.01	Traces	17.9	17.5
C_22_	–	Traces	Traces	Traces	–	Traces
C_24_	–	Traces	–	–	–	–
C_26_	–	Traces	–	–	–	–
Sum	0.67	Traces	0.80	Traces	–	–

Cuticular alcohols: sum of alcohols content in petroleum extract (I), and dichloromethane extract (II)

Internal alcohols: content of alcohols in extract III

Data are presented as the means ± standard deviations of three separate analyses performed on different samples

### Alcohol Composition of Female *L. sericata*

The cuticular lipids of the female contained seven saturated alcohols with even-numbered carbon chains from C_10:0_ to C_22:0_ (Table 5). The alcohol C_18:0_ was present in the highest concentrations (48.8%) (the same observation as for the male lipids). The total cuticular alcohol content in females of *L. sericata* was 0.80 μg/g (a little more than in the males). Only three alcohols (C_14:0_, C_20:0_ and C_22:0_) were found in traces in the internal lipids of females.

### Cholesterol Content in Male and Female *L. sericata*

The cholesterol contents in the cuticular lipids of males and females were 7.6 and 11.4 μg/g of the insect body, respectively (Table 6). Considerably more cholesterol was present in the internal lipids of these insects. The quantities of internal cholesterol in female lipids were double those found in males. The cholesterol was identified on the basis of the characteristic ions (as the trimethylsilyl ether).

**Table 6:** Chemical composition of the cholesterol found in male and female of *Lucilia sericata*

	Content (μg/1 g)	
	Cuticular cholesterol	Internal cholesterol
Male	7.6 ± 0.4	4.9 × 10^2^ ± 0.2 × 10^2^
Female	11.4 ± 0.8	9.7 × 10^2^ ± 0.3 × 10^2^

Cuticular cholesterol: sum of cholesterol content in petroleum extract (I), and dichloromethane extract (II)

Internal cholesterol: content of cholesterol in extract III

Data are presented as the means ± standard deviations of three separate analyses performed on different samples

## Discussion

Free fatty acids of *L. sericata* were present not only in the cuticle; large amounts were also detected in the internal lipids. The efficiency of cuticular and internal lipids extraction differed in *L. sericata* males and females. Amounts of cuticular lipids (mg/g of the insect body) extracted with the use of petroleum ether and dichloromethane from males were 2.1 and 1.1 times higher, respectively, than the amounts of extracts obtained from females. Similarly, amounts of internal lipids extracted from males were 1.3 times higher than analogous extracts obtained from females. The total amounts of extracted lipids (cuticular and internal) comprised 2.99% of male and 2.17% of female wet body weight, respectively. From Gilbert [27] it appears that lipid content in male adults of *Calliphora vicina* (formerly *C. erythrocephala*), closely related to *L. sericata*, and is slightly higher than in females (3.4 vs. 3.3% wet weight). In most insect species the female usually contains more lipids than the male, as a lipid is a most efficient substrate for egg development. However, the reverse may be true for many species and this is especially evident when Lepidoptera are considered [27]. Sexual dimorphism in lipid content in the adult stage was studied in detail in the silk moth *Hyalophora cecropia*. The tissues of *H. cecropia* males contain about five times as much lipid per gram of fresh weight as the tissues of the female. The higher concentration of lipids in the male moth is most likely correlated with mating behavior, since the male moth flies relatively great distances in search of virgin females while the female does only limited flying after emergence. The silk moth male appears to utilize lipid as a primary substrate for flight while female *H. cecropia* converts a large percentage of her endogenous substrate into eggs [27]. In contrast, adults of *L. sericata* utilize carbohydrates as the main source of flight energy [28]. In spite of intensive studies concerning the flight of *L. sericata* and some other Diptera species, no sexual difference in the flight behavior and biochemistry was evidenced [29, 30].

Cuticular fatty acids in male and female extracts made up ca 0.14 and 0.12% of all lipids, respectively. The amounts of FFA in cuticular lipids can vary with respect to sex, stage and living conditions. It has been shown that FFA comprise from 2.04 to 0.50% of the lipids in the exuviae of *Dendrolimus pini* [31] and only trace amounts of FFA were detected in cuticular lipids from nymphs and exuviae of the Silver leaf whitefly, *Bemisia argentifolii* [32], whereas FFA make up 79.40% of the cuticular lipids of *Calliphora vicina* larvae [20]. Cuticular FFA in males and females of *L. sericata* ranged from C_6_ to C_20_; moreover, they consisted of both odd- and even-numbered carbon chains. Similar profiles were identified in closely related *C. vicina* larvae [20]: the fatty acids in this insect ranged from C_5_ to C_20_, although the typical cuticular fatty acids ranged from C_12_ to C_20_. For example, fatty acids in this range were found in lipids from *Frankliniella occidentalis* adults and larvae [33], *Acanthoscelides obtectus* males and females [25], *Liposcelis bostrychophila* [34] and *Fannia canicularis* [35]. In our study, the cuticular fatty acids identified in the adult insects were both saturated and unsaturated. Odd- and even-numbered, saturated and unsaturated fatty acids are typically found in many insect species. The presence of polyunsaturated acids 20:4n-3 and 20:5n-3 in cuticular lipids is rather unusual, although these acids were identified in the lipids of the aquatic insect larvae *Stictochironomus pictulus* [36]. In our work, the females contained these compounds in respective concentrations of 1.6 and 1.0%. Moreover, trace amounts of 20:4n-3 and 20:5n-3 acids were present in the male cuticular lipids. The female extract contained C_7_ and C_20_ acids, which were absent from the male extract. On the other hand, C_17_ acid occurred only in the male extract.

The profiles of predominant cuticular FFA in males and females of *L. sericata* were similar. The predominant components of males and females consisted of 16:0 and 18:0 and also 16:1n-9 and 18:1n-9 fatty acids. Other cuticular fatty acids were present in much smaller quantities. Only four unsaturated fatty acids were present in both males and females. Internal FFA in males and females of *L. sericata* made up 40.4 and 37.7% of all lipids, respectively. Free fatty acids were extracted from both sexes of *L. sericata* with carbon numbers ranging from C_6_ to C_26_, so the internal free fatty acid profiles of both adult insects were similar. In this case, the acids present in the highest concentrations in the internal extract were 16:1n-9, 16:0 and 18:1n-9. The contents (%) of 20:5n-3 and 20:4n-3 fatty acids of both sexes were similar; there was a significant difference between the amounts (μg/g) of these acids (male: 0.56 mg/g of the insect body and female: 0.38 mg/g of the insect body). The female extract contained 7:0, and traces of 10:0 and 14:1n-9 acids, which were absent, from the male extract. 22:0, 24:0 and 26:0 acids were present in the internal lipids, which were absent from the cuticular male and female extracts.

The alcohols present in the cuticular lipids of insects usually have an even number of carbons and are saturated [32, 37–39]. In our study, the cuticular lipids of females of *L. sericata* contained seven even-numbered, saturated alcohols from C_10_ to C_22_, but the cuticular lipids of the males contained only five such compounds, from C_12_ to C_20_. C_10_ and C_22_ alcohols were present only in female cuticular lipids. However, the remaining alcohols were identified in both samples at similar levels. The alcohol present in the highest concentrations in males and females was C_18_ (55.2 and 48.8%, respectively). The alcohols found in our study ranged from C_10_ to C_24_, and similar profiles were identified in *Locusta migratoria migratoriodes* (from C_10_ to C_34_) and *Schistocerca gregaria* (from C_10_ to C_32_) [40]. It is known that the amounts of alcohols in the cuticular lipids of an insect may differ significantly between various species. For example, alcohols made up 42% of all cuticular lipids in pupae of *Heliothis virescens* [37], but only 3 and 4% in nymphs and exuviae of *B. argentifolii* [32]. In our work, the cuticular alcohols in male and female *L. sericata* were <1% of all lipids. Internal lipids of females and males of *L. sericata* respectively contained three (from C_14_ to C_22_) and five (from C_14_ to C_26_) alcohols in trace quantities.

Diverse biological functions of cuticular alcohols have been reported. In the European honey bee, alcohols may protect against parasite attack. Extracts of *Apis mellifera* cocoons containing alcohols of 17–22 carbons induce a strong arrestment response in the mite *Varroa jacobsoni* [41]. A mixture of short chain alcohols, their acetate derivatives, and (*Z*)-11-eicosenol secreted by the sting apparatus of the worker honey bee are components of bee alarm pheromones [42]. The sex pheromones of the three most important tortricids of European vineyards, *Eupoecilia ambiguella*, *Sparganothis pilleriana* and *Lobesia botrana*, have been chemically investigated and found to contain up to 15 different straight-chain acetates and alcohols [43]. The role of alcohols found in *L. sericata* remains unknown.

In cuticular and internal lipids of *L. sericata*, cholesterol was identified on the basis of the characteristic ions (*m*/*z* 129 (100%), 329 (87%), 145 (38%), 121 (36%), 353 (32%) and M^+·^ 458) [44] (as the trimethylsilyl ether). Female lipids contained twice as much cholesterol as male lipids. Internal cholesterol in male and female extracts made up 2 and 5% of all lipids, respectively. The respective quantities of cholesterol obtained from male and female *L. sericata* were 0.49 ± 0.02 and 0.97 ± 0.03 mg/g of the insect body, respectively. There was a tenfold higher concentration of cholesterol in internal lipids. Sterols are minor constituents of cuticular lipids [45]. The cholesterol content in cuticular male and female lipids was 7.6 ± 0.4 and 11.4 ± 0.8 μg/g, respectively. Assuming that sterols are mandatory for egg production and normal embryonic development, a higher concentration of internal cholesterol in *L. sericata* females seems justified. In contrast, the physiological role of cuticular cholesterol remains obscure.

Naturally occurring entomopathogenic fungi are important regulatory factors of insect populations. *Conidiobolus coronatus*, a cosmopolitan soil fungus causing rapid death of susceptible insects, due to the secretion of toxic metabolites [46, 47] was used in current studies. Exposure of *L. sericata* to sporulating fungal colonies resulted in prompt death of both, males and females while larvae and pupae remained unharmed and developed normally. Similarly, the larvae of closely related *C. vicina* showed amazing resistance to *C. coronatus*, while exposure of two lepidopteran larvae, *G. mellonella* and *D. pini*, resulted in their death. Microscopic studies revealed that the conidia of *C. coronatus* did not germinate on the cuticle of *C. vicina* larvae while the cuticles of both lepidopteran larvae were infiltrated by fungal hyphae [47, 48]. The impressive *C. vicina* resistance to fungus is accompanied by a high resistance of the cuticle to degradation by fungal proteases and a high antiproteolytic capacity of insect hemolymph. On the other hand, the immune system of challenged larvae shows very low activity of both, cellular and humoral components. It seems that the significant protection provided by the *C. vicina* cuticle reduces potentially costly defense responses within the hemocoel. Investment in cuticular protection comes at the cost of low phenoloxidase, lysozyme, encapsulation, and phagocytic activities [48]. The cuticular fatty acids profile of *C. vicina* larvae significantly differs from profiles of *D. pini* and *G. mellonella*. Data from in vitro cultures of *C. coronatus* in media supplemented with various fatty acids showed strong fungistatic effects of 18:2n-6, 18:3n-6, 20:1n-3, and 20:0 [21]. It should be pointed out that all these compounds are missing in the cuticular lipids of *L. sericata* adults (traces of 20:0 were detected in females only). On the other hand, 16:1n-9 stimulating fungal virulence [21] is present at high concentrations in both males and females (10.3 and 15.2% of all cuticular lipids, respectively) suggesting that this compound may be responsible for the prompt death of flies exposed to *C. coronatus* colonies. Analysis of cuticular lipids of *L. sericata* larvae and pupae (currently underway in our laboratories) should provide information on whether the high susceptibility of *L. sericata* adults to fungal infection opposed to the resistance of the larvae and pupae is linked with diverse cuticular lipid profiles. Work on the effects of new compounds found in adults of *L. sericata* on the pathogenicity potential of this entomopathogen is in progress. Knowledge of the role of cuticular lipids in fungal interaction with the insect host can be expected to contribute to a better understanding of the nature of fungal pathogenicity.

## Abbreviations

SAM: Sabouraud agar medium
TIC: Total ion current
SIM: Single ion monitoring
EI: Electron impact
BSTFA: *N*,*O*-Bis(trimethylsilyl) trifluoroacetamide
TMCS: Trimethylchlorosilane
M^+·^: Molecular ion
TMSi: Trimethylsilyl derivatives
FFA: Free fatty acids
7-T, 23:1: 7-Tricosene
7-P, 25:1: 7-Pentacosene
7,11-HD, 27:2: 7,11-Heptacosadiene
7,11-ND, 29:2: 7,11-Nonacosadiene
